# Comparison of the chemical profiles and inflammatory mediator-inhibitory effects of three *Siegesbeckia* herbs used as Herba Siegesbeckiae (*Xixiancao*)

**DOI:** 10.1186/s12906-018-2205-x

**Published:** 2018-05-02

**Authors:** Hui Guo, Yi Zhang, Brian Chi-Yan Cheng, Mei-Yuk Lau, Xiu-Qiong Fu, Ting Li, Tao Su, Pei-Li Zhu, Yuen-Cheung Chan, Anfernee Kai-Wing Tse, Tao Yi, Hu-Biao Chen, Zhi-Ling Yu

**Affiliations:** 1 Centre for Cancer and Inflammation Research, School of Chinese Medicine, Hong Kong Baptist University, Kowloon Tong, Hong Kong, SAR China; 2 Research and Development Centre for Natural Health Products, HKBU Shenzhen Research Institute and Continuing Education, Shenzhen, China; 3 Consun Chinese Medicines Research Centre for Renal Diseases, Hong Kong Baptist University, Hong Kong, SAR China

**Keywords:** Herba Siegesbeckiae, *Siegesbeckia pubescens*, *Siegesbeckia orientalis*, *Siegesbeckia glabrescens*, Comparison, Chemical profiles, Inflammatory mediator

## Abstract

**Background:**

Herba Siegesbeckiae (HS, *Xixiancao* in Chinese) is a commonly used traditional Chinese medicinal herb for soothing joints. In ancient materia medica books, HS is recorded to be the aerial part of *Siegesbeckia pubescens* Makino (SP) which is also the only origin of HS in the 1963 edition of the Chinese Pharmacopeia (ChP). The aerial parts of *Siegesbeckia orientalis* L. (SO) and *Siegesbeckia glabrescens* Makino (SG) have been included as two additional origins for HS in each edition of ChP since 1977. However, chemical and pharmacological comparisons among these three species have not been conducted.

**Methods:**

An HPLC with diode array detector (HPLC-DAD) method combined with similarity analysis, hierarchical cluster analysis (HCA) and principal component analysis (PCA) was developed for comparing the fingerprint chromatograms of the three species. The inhibitory effects of the three species on NO production and IL-6 secretion in LPS-stimulated RAW264.7 macrophages were compared.

**Results:**

Fingerprint chromatograms of the three species showed different profiles, but had 13 common peaks. Results from HCA and PCA of the common peaks demonstrated that all 14 herbal samples of the three species tended to be grouped and separated species dependently. The extents of inhibition on NO production and IL-6 secretion of the three species were different, with SG being the most and SP the least potent.

**Conclusions:**

Both chemical profiles and inflammatory mediator-inhibitory effects of the three species were different. These findings provide a chemical and pharmacological basis for determining whether the three species can all serve as the origins of HS.

## Background

Chinese medicines (CMs) have been widely used for thousands of years in many Asian countries, such as China, Korea, Japan, etc. However, CMs have not been universally accepted due to insufficient safety and efficacy evidence to meet the modern standards worldwide [1–3]. Some CMs even have more than one natural origin. Herba Siegesbeckiae (HS, *Xixiancao* in Chinese) is one of the widely used CMs and prescribed by Chinese doctors to treat inflammatory joint diseases such as arthritis and rheumatoid arthritis (RA) [4–7]. In the 1963 edition of the Chinese Pharmacopeia (ChP), only the aerial part of *Siegesbeckia pubescens* Makino (SP) was recorded to be the origin of HS, which was consistent with the species used in ancient time [8]. While, two more *Siegesbeckia* species, *Siegesbeckia orientalis* L. (SO) and *Siegesbeckia glabrescens* Makino (SG) have been included as additional origins for HS in each edition of ChP since 1977 for expanding herb material sources. Previous chemical and pharmacological studies about HS mainly focused on individual species [9–13]. To our knowledge, very little effort has been made to investigate the chemical and pharmacological differences among these three species, although people questioned whether the three species can all be the origins of HS. In the present study, the chemical profiles and the inflammatory mediator-inhibitory effects of the three *Siegesbeckia* species were compared.

## Methods

### Reagents and materials

Lipopolysaccharide (LPS) from *Escherichia coli* O55:B5, dimethyl sulphoxide (DMSO), 3-(4,5-dimethylthiazol-2-yl)-2,5-diphenyltetrazolium bromide (MTT) and Griess reagent were obtained from Sigma Chemicals Ltd. (St. Louis, MO, USA). Penicillin, streptomycin, Dulbecco’s Modified Eagle Medium (DMEM) and foetal bovine serum (FBS) were purchased from Hyclone (Logan, UT, USA). Acetonitrile (ACN, HPLC grade) and phosphoric acid (PA) were obtained from RCI Labscan Limited (Thailand). A Milli-Q system (Millipore, MA, USA) was used to prepare purified water for HPLC analysis. Analytical grade methanol and ethanol (absolute) from Merck (Darmstadt, Germany) were used for sample preparation. Other materials used in bioassays were from Life Technologies Inc. (GIBICO, USA).

### Herbal material preparation

Plants of the genus *Siegesbeckia* (Compositae) are annual herbs widely distributed in China [14, 15]. Fourteen batches of raw HS were collected from different geographical regions of China from June 4 to June 15 in the same year (Table 1). All batches were authenticated by Professor Hubiao Chen from HKBU. Voucher specimens were deposited at the School of Chinese Medicine, HKBU. The collected samples were cleaned and dried in a hot air oven at 45 °C for 12 h. The dried samples were grounded to a fine powder and passed through 60 sieve mesh and stored in airtight containers until use.

**Table 1:** Herbal sample information

No.	Species	Sources (Geographical location)	GPS coordinates	Collection time	Deposition numbers
1	*S. pubescens*	Shenyang, Liaoning	E: 123.43° N: 41.81°	2013–6-14	SP-1
2	*S. pubescens*	Changchun, Jilin	E: 125.33° N: 43.82°	2013–6-15	SP-2
3	*S. pubescens*	Tonghua, Jilin	E: 125.94° N: 41.73°	2013–6-15	SP-3
4	*S. pubescens*	Chengde Hebei	E: 117.96° N: 40.95°	2013–6-8	SP-4
5	*S. pubescens*	Zunhua, Hebei	E: 117.97° N: 40.19°	2013–6-8	SP-5
6	*S. pubescens*	Nanning, Guangxi	E: 108.37° N: 22.82°	2013–6-4	SP-6
7	*S. orientalis*	Changde,Hunan	E: 111.70° N: 29.03°	2013–6-7	SO-1
8	*S. orientalis*	Anguo, Hebei	E: 115.33° N: 38.42°	2013–6-7	SO-2
9	*S. orientalis*	Cangzhou, Hebei	E: 116.84° N: 38.31°	2013–6-8	SO-3
10	*S. orientalis*	Changchun, Jilin	E: 125.33° N: 43.82°	2013–6-15	SO-4
11	*S. glabrescens*	Jinhua, Zhejiang	E: 119.65° N: 29.08°	2013–6-6	SG-1
12	*S. glabrescens*	Lishui, Zhejiang	E: 119.92° N: 28.47°	2013–6-6	SG-2
13	*S. glabrescens*	Nanchang, Jiangxi	E: 115.86° N: 28.68°	2013–6-5	SG-3
14	*S. glabrescens*	Guangzhou, Guangdong	E: 113.27° N: 23.13°	2013–6-4	SG-4

To prepare extracts for HPLC analyses, each herb powder (0.3 g) was accurately weighed and added to 10 mL methanol. The mixture was precisely weighed and then sonicated for 60 min (CREST 1875HTAG ultrasonicator, U.S.A.). After the mixture cooled to the room temperature, methanol was added to compensate for the lost weight during the extraction, and then the extract was filtered with a syringe filter (0.45 μm, Millipore). The filtrate (10 μL) was injected for HPLC analysis.

To prepare extracts for bioassays, three herbal samples with the highest degree of similarity, in similarity analyses, from each species were selected as the representatives of SO, SP and SG. HS is traditionally used in the forms of decoction (extraction using water) and pill made from herb powder. Its bioactive components may comprise polar and/or nonpolar compounds. Thus, three extracts were prepared from each representative sample by using three different solvents (water, 50 and 95% ethanol). Each accurately weighed sample (10 g) was immersed in the solvent at a sample/solvent ratio of 1:10 (w/v) for 1 h, and then refluxed three times for 1 h each time. The extracts were filtered and combined after cooling; and the filtrate and washings were combined and then concentrated by rotary evaporation under reduced pressure to remove the solvent. The concentrated extracts were rapidly frozen at − 80 °C, and then dried in a freeze-dryer (Virtis freeze mobile, Virtis Co., Gardiner, USA). For the water extraction, the yields of SO, SP, and SG were 19.68, 16.37 and 17.29%, respectively. For the 50% ethanol extraction, the yields of SO, SP, and SG were 14.43, 11.25 and 12.12%, respectively. For the 95% ethanol extraction, the yields of SO, SP, and SG were 7.53, 5.19 and 5.24%, respectively. The extracts were stored at 4 °C until further use.

### HPLC analysis

HPLC fingerprint analysis was conducted on an Agilent 1260 series HPLC-DAD system including binary pump (G1312B), vacuum degasser (G1322A), autosampler (G1329B), column compartment (G1316A) and diode array detector (G4212A). All separations were performed on an Alltima™ C-18 analytical column (250 mm × 4.6 mm I.D., 5 μm) and protected with an Alltima C-18 guard-column (12.5 mm × 4.6 mm I.D., 5 μm). A linear gradient system consisted of A (0.1% PA aqueous solution: PA, analytical grade, RCI Labscan Limited) and B (ACN, HPLC grade, RCI Labscan Limited). The gradient elution profile was as follows: 0–5 min, 2% B; 5–25 min, 2–20% B; 25–45 min, 20–25% B; 45–60 min, 25–40% B; 60–80 min, 40–70% B; 80–90 min, 70–90% B; 90–100 min, 90% B. Each run was followed by an equilibration period of 15 min. The flow rate was maintained at 1.0 mL/min and the column temperature was set at 30 °C. The Chromatograms were monitored with the DAD detector at a wavelength of 215 nm. 10 μL per sample was injected for analysis.

### Method validation

The developed HPLC method was validated by testing precision, repeatability and stability. The relative standard deviation (RSD) of six repeated runs of one sample was used to evaluate the precision. To test the repeatability of the method, six replicate samples prepared independently were injected for HPLC analyses. For the stability test, a sample was analyzed 0, 12, 24, 36 and 48 h after the completion of sample preparation.

### Chemometric methods

(1) Similarity analysis (SA). Similarity analysis was performed to determine the degree of similarity or dissimilarity of chromatograms using the software named Similarity Evaluation System for Chromatographic Fingerprint of TCM (Version 2004A; National Committee of Pharmacopoeia, China). A simulative median chromatogram (SMC) was generated by this software as a representative reference chromatogram (RC) for all or designated samples. Then, similarity value of the chromatogram of each sample was calculated against RC via the same software. Peaks existing in all sample chromatograms were assigned as common peaks. In the chromatograms, thirteen characteristic common peaks were selected and marked; and the peak of kirenol was designated as the reference peak. (2) Hierarchical clustering analysis (HCA). The SPSS software (SPSS for Windows 11.5, SPSS Inc., USA) was utilized to perform the hierarchical clustering analysis of the common peaks of samples 1–14. Specific method (average linkage between groups) and measurement (Pearson correlation) in the software were selected to run the analysis. (3) Principal component analysis (PCA). To evaluate whether the fingerprint profiles can effectively distinguish HS from different species, PCA (a typical exploratory analysis), using the software SIMCA 13 (Umetrics, Sweden), was chosen to monitor the outline of all data in multivariate analyses. In this study, the score plot was defined by the interrelation between the first three principal components for visualisation of the data matrix.

### Macrophage cell culture

The murine macrophage cell line RAW267.4 was obtained from the American Type Culture Collection (Manassa, VA, USA) and maintained in DMEM medium supplemented with 5% heat-inactivated FBS and 1% antibiotics of penicillin/streptomycin at 37 °C under 5% CO_2._

### Cell viability assay

Cell viability was evaluated using the MTT assay. RAW264.7 cells were seeded on 96-well plates (5 × 10^3^ cells/well) for 24 h. The cells were treated with indicated concentrations of an extract for 1 h, and then treated at the presence or absence of LPS (100 ng/mL) for 24 h. After that, the medium was then discarded and the cells were incubated with the MTT solution (0.5 mg/mL) for another 3 h. The supernatant was then removed and the remaining formazan crystals were dissolved in 100 μL DMSO. The optical density was measured at 570 nm using a microplate spectrophotometer [16].

### Determination of nitric oxide (NO) production

The RAW264.7 cells were seeded on 24-well culture plates (2 × 10^5^ cells/well) for 24 h. Various concentrations of each extract were prepared. After 1 h pre-treatment with indicated concentrations of an extract, the cells were then treated with or without LPS (100 ng/mL) for another 24 h. NO production was determined by measuring the accumulated nitrite in the culture medium with Griess reagent [6]. Absorbance at 540 nm was measured using a microplate spectrophotometer (BD, Bioscience USA). The EC_50_ value (the concentration at which NO production was inhibited by 50%) was determined with the curve-fitting software GraphPad Prism 5.0 (GraphPad Software, San Diego, CA).

### Enzyme-linked immunosorbent assay (ELISA)

ELISA kit purchased from Invitrogen (Carlsbad, CA, USA) was used to determine IL-6 production in the culture medium. RAW 264.7 macrophages were treated as described in the above section. The determination of IL-6 in the medium was conducted following the manufacturer’s instructions [17].

### Statistical analysis

Results of bioassays were presented as mean ± SD. Statistical significance was determined using one-way ANOVA followed by Tukey’s multiple comparisons test using GraphPad Prism 5.0. *p* < 0.05 was considered to be statistically significant.

## Results

### HPLC method development

Sample pretreatment conditions were first optimized by investigating the effect of extracting solvents on extraction efficiencies for chromatographic peaks. Different HS extracts were prepared using various extracting solvents including methanol, ethanol, 50% methanol, 50% ethanol and water. Results showed that HS extracted using methanol exhibited the largest number of chromatographic peaks and the largest peak area (data not shown), therefore methanol was chosen as the extracting solvent in this study. Two sample extracting methods, ultrasonic extraction and reflux extraction were then compared. Comparable HPLC peak numbers and intensities were achieved with the two methods. Ultrasonic extraction, which is easier for operation, was adopted as the extraction method in the experiment. Besides, extraction durations (including 15, 30, 60 and 120 min) under ultrasonication were also investigated. Results showed that 60 min was sufficient for ideal extraction.

Optimization of HPLC condition was done through investigating the influence of the mobile phase and detection wavelength. Results showed that acetonitrile with 0.1% (v/v) phosphoric acid had the best peak shapes and baseline resolution. In order to obtain a sufficiently large number of detectable peaks on HPLC chromatogram, the detection wavelength was selected by a DAD full wavelength scan (190–400 nm); and 215 nm was selected as detection wavelength. The final method was described in details in the Methods section.

### Method validation

Diterpenoids are reported to be the major bioactive components of HS [15, 16]. Kirenol, a diterpenoid, is the chemical marker for the quality control of HS in ChP. Thus, Peak 6 (kirenol) at retention time 54.3 min with a moderate retention time and peak area was chosen as the reference peak for fingerprint calculation. The relative retention time (RRT) and relative peak area (RPA) generated with respect to the reference peak were used to distinguish peaks and assess the consistency of peaks’ intensities in chromatograms, respectively.

For the HPLC method validation, the RSD of the RRT and RPA of each common peak were used as the appraise indexes. In repeatability tests, RSD values for RRT and RPA were ≤ 1.95 and 1.21%, respectively, indicating high reproducibility of the method. In stability tests, RSD values were ≤ 1.75 and 0.97% for RRT and RPA, respectively, indicating that the sample solution was stable within 48 h. In precision tests, RSD values were ≤ 1.03% for RPT and 0.85% for RRA, indicating that the method was reliable. These data suggest that the HPLC method is suitable for analysis of HS samples.

### HPLC fingerprint chromatograms of SO, SP and SG

**Fig. 1:**
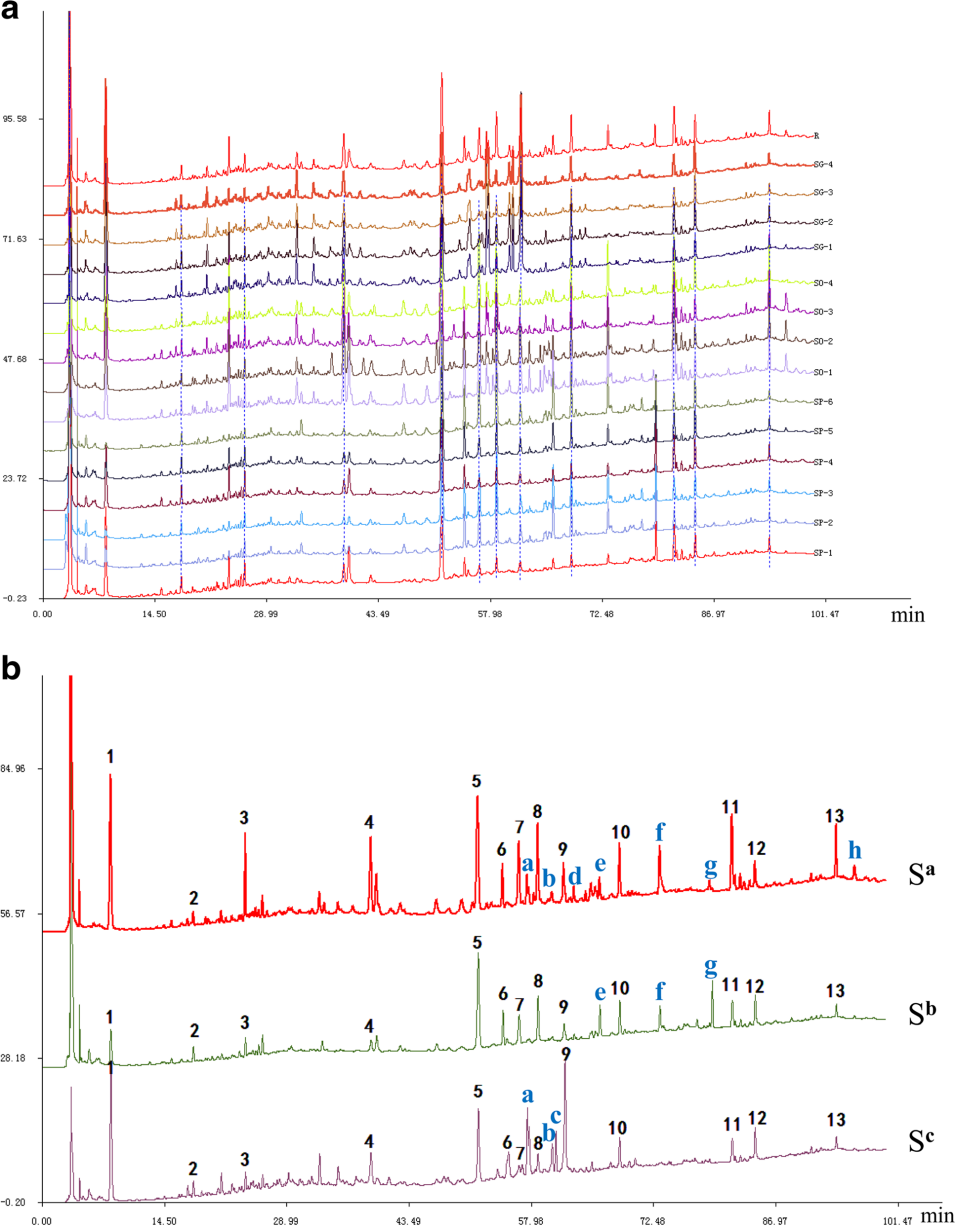
Results of chemical profiles. **a** Overlaid HPLC chromatograms of samples 1–14. **b** Simulative median chromatograms (SMC) for each species. S^a^, SMC for all SO samples; S^b^, SMC for all SP samples; S^c^, SMC for all SG samples. The peaks marked with 1–13 in the SMCs were the common peaks for all 14 samples, and peaks marked with letters were the unique peaks for individual species or unique ones in two of the three species

To compare chemical profiles of the three species, the validated HPLC method was used to obtain chromatograms for all the 14 collected samples. Results were shown in Fig. 1a. The 14 obtained chromatograms were compared using the similarity evaluation software, and the values of similarity were calculated (Table 2). When the SMC for samples of the same species was used as RC (default similarity value = 1.000), similarity values of corresponding samples were all more than 0.950 (Table 2 Similarities^a^ Column), indicating the consistence in chemical profiles of samples from the same species. When SMC for all 14 samples were used as RC (Table 2 Similarities^b^ Column), the similarity of SP and SO samples posed relatively high values of 0.932 to 0.980; while, SG samples demonstrated relatively low values (less than 0.8)**,** suggesting that the chemical profiles of SP and SO were similar, while distinct from that of SG**.** Samples 1, 7 and 11, which showed the maximum value of similarity in each species were selected as the representative samples for SO, SP and SG, respectively. Extracts of these three samples were used for bioassays.

**Table 2:** Results of similarities

Species	No.	Similarities^a^	Similarities^b^
SP	1	0.980	0.975
	2	0.985	0.943
	3	0.977	0.939
	4	0.985	0.973
	5	0.980	0.949
	6	0.954	0.932
SO	7	0.995	0.980
	8	0.986	0.969
	9	0.955	0.963
	10	0.986	0.967
SG	11	0.994	0.794
	12	0.959	0.687
	13	0.982	0.738
	14	0.994	0.754

^a^RC for each species (SP, SO and SG) was used in the similarity calculation

^b^RC for all 14 samples was used in the similarity calculation

The SMCs of the three species were shown in Fig. 1b. They were similar to each other; but there were virtually unmatched peaks, indicating the difference among species. The similarity among the three species was demonstrated by 13 common peaks in the SMCs. As for the difference, peaks d and h presented solely in SO samples, peak c was unique to SG samples, peaks a and b were only in SO and SG samples, and peaks e, f and g could only be found in SO and SP samples.

### Chemometric analyses

**Fig. 2:**
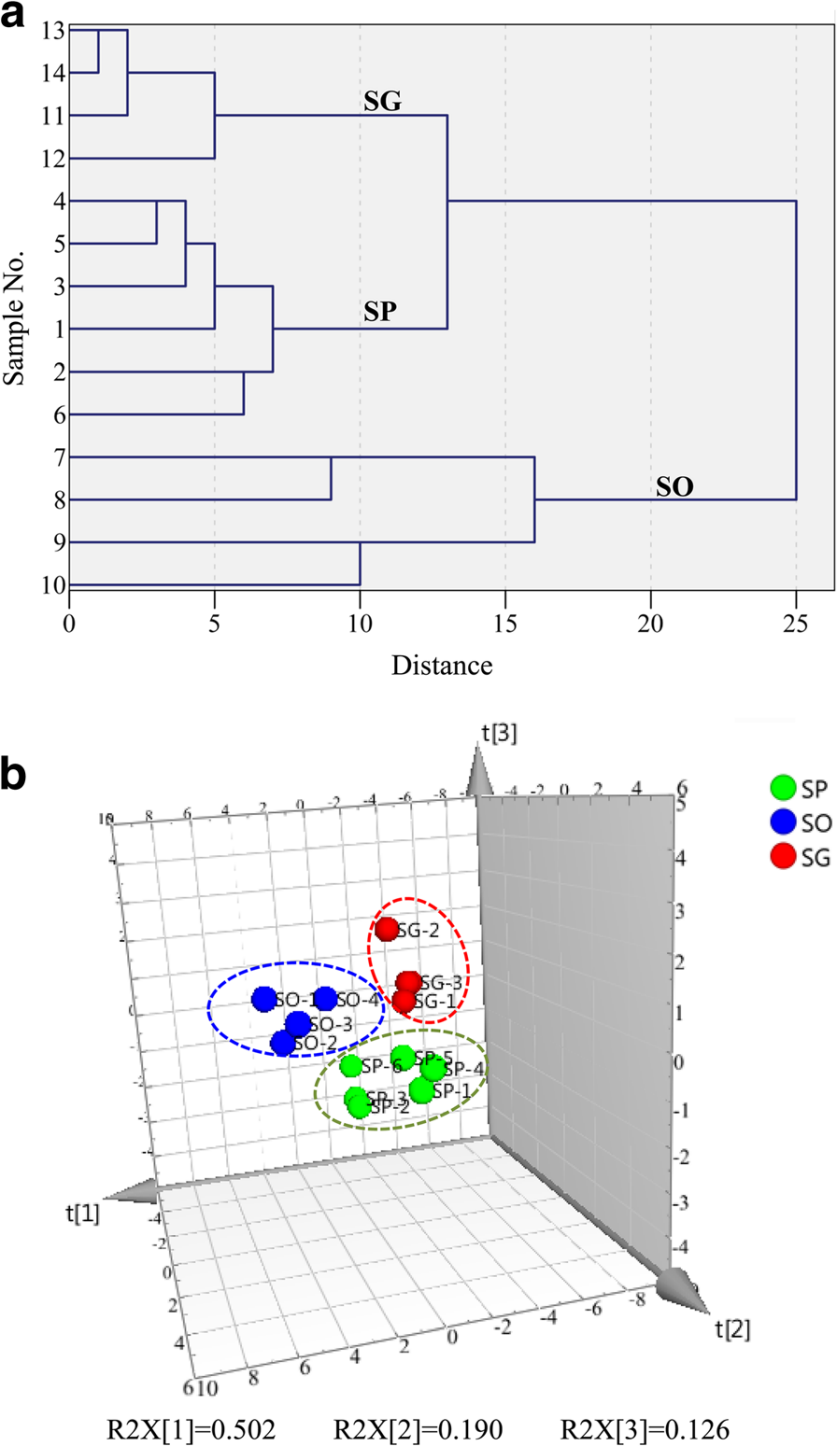
Chemometric analyses of common peaks’ intensities of samples 1–14. **a** Results of hierarchical clustering analysis. **b** Results of principal component analysis. The green, blue and red balls represent SP, SO and SG, respectively

Hierarchical cluster analysis (Fig. 2a) of the 13 common peaks showed that all the 14 herbal samples were clearly grouped into three categories which were consistent with their classifications of species. For the PCA analyses (Fig. 2b), initial eigenvalues were obtained using the SIMCA software. The cumulative percent variance (CPV) of the first three principal variables was calculated as 81.8% which meets the requirements of CPV (more than 70~ 85%) for PCA analysis [18–20]. The PCA results were shown in a three-dimensional (3D) score plot. In the graphic, it was evident that the samples were grouped species dependently.

### Bioassays for the representative samples for SO, SP and SG

**Fig. 3:**
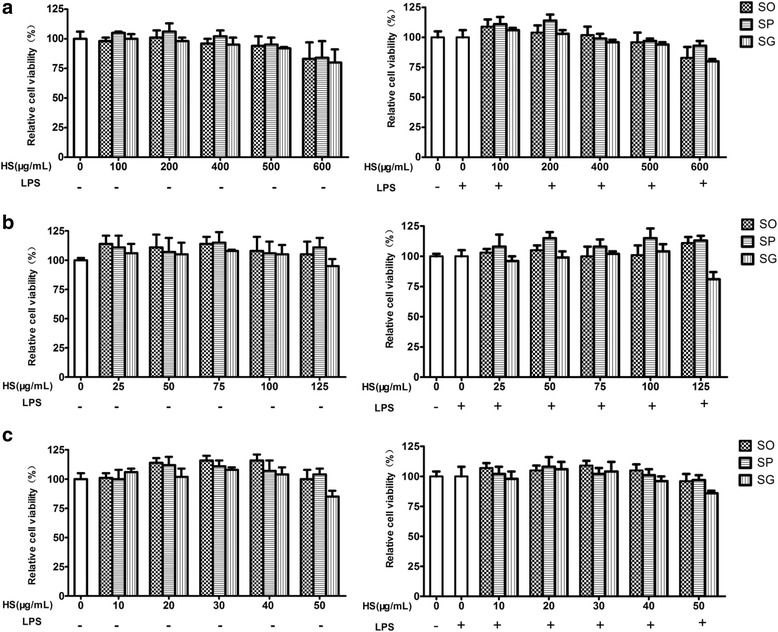
Effects of HS extracts (SO, SP and SG) on the viability of RAW 264.7 macrophages. **a** Water extracts; **b** 50% ethanol extracts; **c** 95% ethanol extracts. The cells were incubated with the indicated concentrations of HS extracts for 24 h with (right panel) or without LPS (left panel). Cell viability was determined by the MTT assay. Results were expressed as the percentage against respective control

**Fig. 4:**
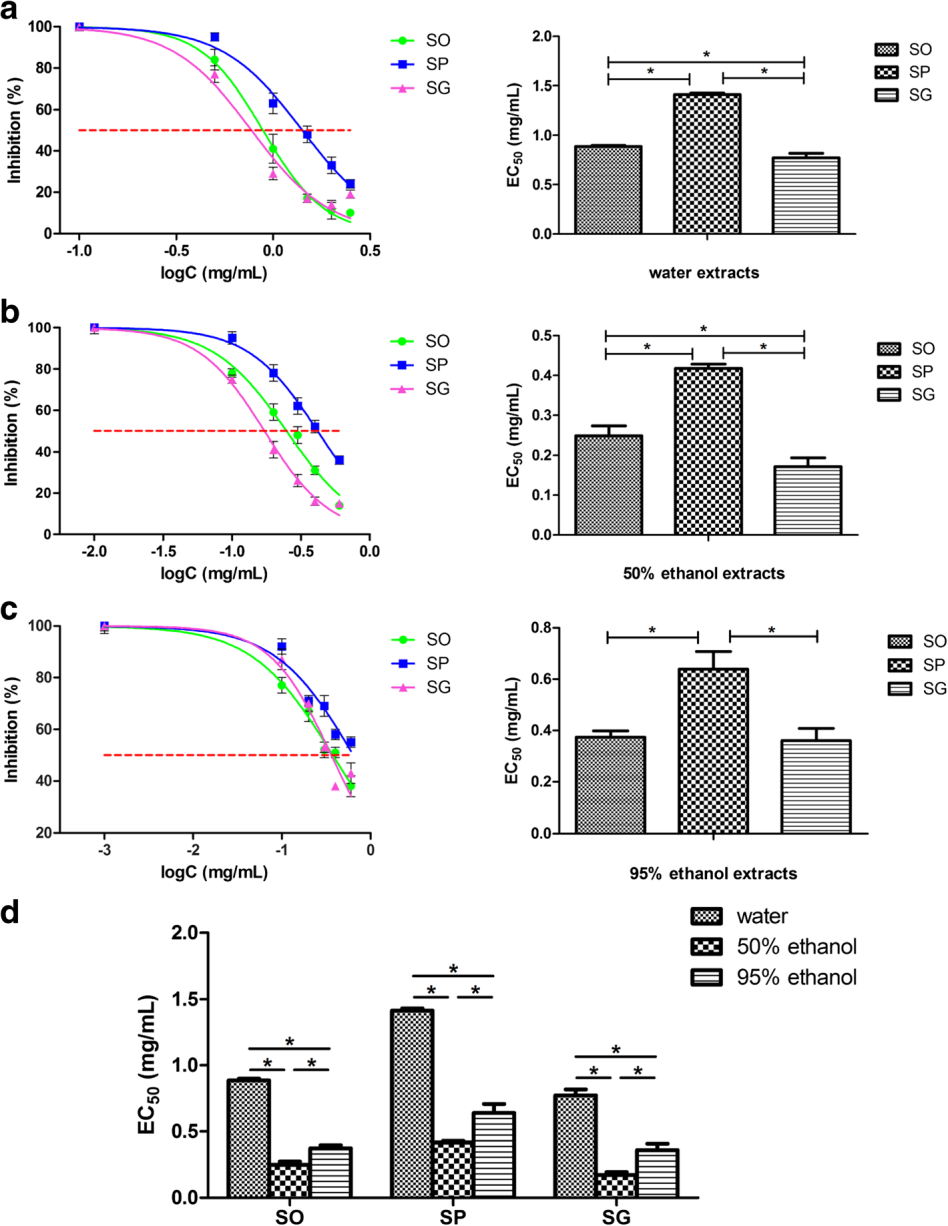
Bioactivity comparison results of SO, SP and SG. **a** Effects of water extracts on NO production. **b** Effects of 50% ethanol extracts on NO production. **c** Effects of 95% ethanol extracts on NO production. RAW 264.7 cells were treated with various concentrations of individual extract for 1 h and then stimulated with 100 ng/mL LPS for 24 h. NO production was indicated by the level of nitrite in the supernatant measured with the Griess reagent. Inhibition rate (%) of an extract was calculated against LPS alone-treated cells. EC_50_ was calculated using the curve-fitting software GraphPad Prism 5.0. **d** Summary of EC_50_ values shown in (**a**), (**b**) and (**c**). Data are shown as mean ± SD from tree independent experiments. ^#^*p* < 0.05, *p < 0.01

**Fig. 5:**
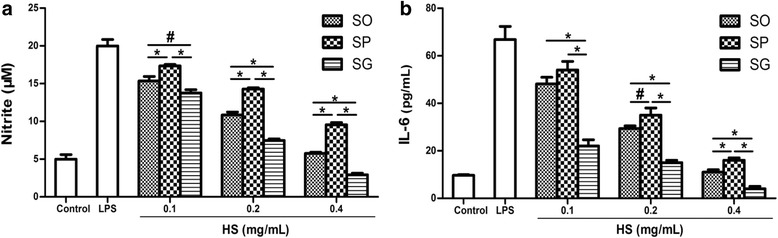
Dose-dependent effects of 50% ethanol HS (SO, SP and SG) extracts on production of NO and IL-6. **a** Effects on NO production. **b** Effects on IL-6 production. Cells were treated with three concentrations (equivalent to 0.1, 0.2 and 0.4 mg herb/mL) of individual HS extract for 1 h and then treated with 100 ng/mL LPS for another 24 h. NO production was determined as in Fig. 4. IL-6 production was determined by ELISA. Data are shown as mean ± SD from tree independent experiments. ^#^*p* < 0.05, *p < 0.01

Three extracts were prepared from each representative sample by using three different solvents (water, 50 and 95% ethanol). To determine the sub-cytotoxic concentrations of the three representative samples, MTT assays were conducted. In the presence or absence of 100 ng/mL of LPS, the viability of RAW264.7 cells remained unaffected (≥ 90% viability) after a 24-h incubation with up to 500 μg/mL of each water extract (Fig. 3a), 100 μg/mL of each 50% ethanol extract (Fig. 3b) and 40 μg/mL of each 95% ethanol extract (Fig. 3c). EC_50_ values of water extracts of SO, SP and SG were 0.86, 1.41, 0.77 mg herb/mL (calculated based on the yield of each extract), respectively (Fig. 4a). EC_50_ values of 50% ethanol extracting samples were 0.25, 0.42, 0.17 mg herb/mL for SO, SP and SG, respectively (Fig. 4b). EC_50_ values of 95% ethanol extracts of SO, SP and SG were 0.37, 0.70, 0.36 mg herb/mL, respectively (Fig. 4c). To neatly illustrate the potency of the extracts prepared using different solvents, the EC_50_ values were rearranged and shown in Fig. 4d. The extract prepared with 50% ethanol showed the most potent inhibitory effect on NO production; and the water extract showed the least potent inhibitory effect. In every solvent prepared extracts for different species, EC_50_ value of SG was the lowest, and SP the highest, suggesting that SG had the most and SP the least potent inhibitory effect on NO production. We further investigated the effects of 50% ethanol extracts of the three species on the production of pro-inflammatory cytokines NO and IL-6 in LPS-stimulated RAW264.7 cells. Stimulation with LPS resulted in a marked increase in the production of NO and IL-6 when compared with the unstimulated control. Each of the three species concentration-dependently inhibited NO (Fig. 5a) and IL-6 (Fig. 5b) productions; with SG being the most potent, SP the least potent species.

## Discussion

Currently, it is a great challenge for CMs to escalate their role in the mainstream pharmaceutical market for several not addressed fundamental issues such as supply, quality control, safety and efficacy [21]. The multi-origin issue greatly affects the stability and homogeneity of CM quality, as well as the clinical efficacy and safety.

The multi-origin Chinese medicinal herb HS was first documented in Newly Revised Materia Medica (*Xinxiu Bencao*) issued in A.D. 659 in the Tang Dynasty of China. In ancient materia medica books, this herb was titled various names such as Pig Pungent Weed, Dog Pungent Weed, Tiger Pungent Weed, *Huoqian*, etc. Li Shizhen, a famous traditional Chinese medicine practitioner in the Ming Dynast, identified HS from other misused herbs and recorded his findings in his book Compendium of Materia Medica (*Bencao Gangmu*). Based on Li Shizhen's morphological description of the plant, modern botanists determined SP to be the origin of HS [8]. The other two species SO and SG have been included as the origins of HS without any supporting evidence. It is still a question whether SO, SP and SG can all be the origins of HS.

In this study, using a validated HPLC method we analyzed the chemical profiles of collected HS samples of the three species. The unmatched peaks in SMCs of the three species indicate differences among species. Besides the visually different peaks of the three species, chemometric analyses have demonstrated that there is species-dependent variance in the intensities of common peaks of the three species. Our chemical assays suggest that the three species are chemically different.

HS is used for treating inflammatory diseases and has anti-inflammatory pharmacological effects. Of various pro-inflammatory mediators, NO plays an essential role in the pathogenesis of inflammation [22, 23]. LPS, a component of the cell walls of gram-negative bacteria, is one of the most powerful activators of macrophages and induces the production of pro-inflammatory mediators including NO [24, 25]. Therefore, EC_50_ value for inhibiting NO production in LPS-stimulated RAW 264.7 macrophages was used as an index for assessing the bioactivities of the three species. Bioassay results showed that the inhibitory effects of the three species on inflammatory mediators are different; with SG being the most potent, SP the least potent.

In addition to the anti-inflammatory action, HS possesses various bioactivities such as anticancer [26], anti-hypertension [27], anti-thrombus [28] and anti-microbial [29] activities. We have found that the chemical profiles of the three species are different. It is warranted to explore which components are responsible for each of the activities of this herb.

## Conclusions

In this study, a simple and reliable HPLC method, for the first time, was developed and validated for comparison of chemical profiles among three *Siegesbeckia* species. We used the developed HPLC method combined with chemometric analyses (including SA, PCA and HCA) to obtain and analyse chemical fingerprints of HS samples from the three species. Based on the results of chemical analyses, representative sample for each species was selected to conduct bioassays. We, for the first time, found that SO, SP and SG were different in their chemical profiles and inflammatory mediator-inhibitory effects. This study provides a chemical and pharmacological basis for determining whether all the three species can be equivalently used as HS. To make the decision, further studies are needed. In addition, the bioassay results of this work further provide pharmacological justification for the clinical use of HS in inflammatory diseases.

## Abbreviations

DMSO: Dimethylsulphoxide
HCA: Hierarchical cluster analysis
HPLC-DAD: High-performance liquid chromatography with diode array detector
HS: Herba Siegesbeckiae
IL: Inter-leukin
LPS: Lipopolysaccharide
MTT: Methylthiazolyltetrazolium
NO: Nitric oxide
PCA: Principal component analysis
RC: Reference chromatogram
SA: Similarity analysis
SG: *Siegesbeckia glabrescens* Makino
SMC: Simulative median chromatogram
SO: *Siegesbeckia orientalis* L.
SP: *Siegesbeckia pubescens* Makino
